# *Oxytropis ikhbogdicus* (Section *Mesogaea*, Fabaceae), A New Species From Mongolia Based on Morphological and Molecular Analyses

**DOI:** 10.1002/ece3.73803

**Published:** 2026-06-10

**Authors:** Dariganga Munkhtulga, Shukherdorj Baasanmunkh, Nudkhuu Nyamgerel, Batlai Oyuntsetseg, Zagarjav Tsegmed, Dong Pil Jin, Serik Kubentayev, Gun‐Aajav Bayarmaa, Hyeok Jae Choi

**Affiliations:** ^1^ Department of Biology and Microbiology Changwon National University Changwon South Korea; ^2^ Department of Biology, School of Arts and Sciences National University of Mongolia Ulaanbaatar Mongolia; ^3^ Sejong National Arboretum Sejong South Korea; ^4^ Laboratory of NatureLAB Astana International University Astana Kazakhstan

**Keywords:** *Mesogaea*, molecular phylogenetics, morphology, *Oxytropis*

## Abstract

*Oxytropis ikhbogdicus,* a new species endemic to Mongolia, belonging to section *Mesogaea* (Fabaceae), is described and illustrated. The new species was resolved by phylogenetic analysis based on the complete plastome genome, as well as morphological characteristics. Morphologically, the new species closely resembles *O. kansuensis*, *O. ochroleuca* and *O. ochrocephala* within the section *Mesogaea*; but it is distinguished by stems with 1 or 2 conspicuous internodes (vs. stems with 2–5 conspicuous internodes in *O. kansuensis*, *O. ochroleuca*, and *O. ochrocephala* ); calyx campanulate, 6–7 mm long (vs. campanulate, 6.5–11.5 mm long in *O. kansuensis*; broadly cylindric, 7–8 mm long in *O. ochroleuca*; cylindric, 6–8 mm long in *O. ochrocephala* ); and standard 8.5–9.0 mm long, lamina rhombic‐oblanceolate, apex rounded to emarginate (vs. 10–17 mm long, lamina ovate, apex emarginate in *O. kansuensis*; 12–16 mm long, lamina orbicular‐ovate, apex 2‐lobed in *O. ochroleuca*; 10–17 mm long, lamina broadly obovate, apex emarginate in *O. ochrocephala* ). Additionally, *O. ikhbogdicus* is geographically distinct from other morphologically similar species, found on Altai Mountain in Mongolia, while *O. ochroleuca* occurs in Tien Shan in China, Kazakhstan, and Kyrgyzstan, *O. kansuensis* and *O. ochrocephala* occur Qinghai‐Tibetan Plateau in China.

## Graphical abstract

*Oxytropis ikhbogdicus*, a new species endemic to Mongolia, is described and illustrated. The new species was confirmed by phylogenetic analysis based on the complete plastome genome and combined nuclear ribosome internal transcribed spacer (ITS), psbA‐trnH, and trnF‐L sequence data as well as morphological characteristics.

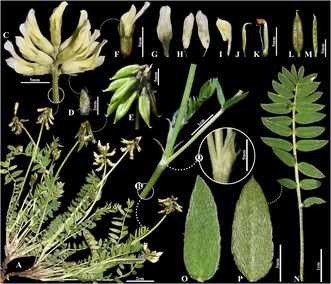

## 1 Introduction

*Oxytropis* L., comprising more than 600 species, is one of the largest and most diverse genera in the Fabaceae (Malyshev 2008; POWO 2025). This genus extends across the subarctic and temperate regions of the Northern Hemisphere (Malyshev 2008; POWO 2025). Many *Oxytropis* species are alpine, with Central Asia recognized as the center of diversity, with approximately 155–170 species (Malyshev 2008; Baasanmunkh et al. 2022; Turdiev et al. 2025). Molecular dating of *Oxytropis* suggests that the genus originated ca. 5.6 million years ago at the Miocene–Pliocene boundary, which roughly coincides with the time of the evolution of ancient species of the genus *Astragalus* L. in South Siberia (Polozhii 2003; Shavvon et al. 2017). *Oxytropis* was first distinguished from *Astragalus* by De Candolle (1802) based on the keel beak and pod septum shape characteristics.

Internal transcribed spacer (ITS) and plastid markers (*mat*K, *trn*L–F, *psb*A–*trn*H) revealed that the genus is monophyletic (Archambault and Strömvik 2012; Shavvon et al. 2017; Kholina et al. 2021). However, many of its traditionally morphologically defined subgenera and sections are not monophyletic based on molecular data, reflecting extensive morphological convergence and parallel evolution across arid and alpine environments (Kholina et al. 2021; Sandanov et al. 2023). With the development of high‐throughput sequencing technology, chloroplast genomes have been widely utilized in plant evolutionary studies, species identification, genetic diversity analysis, chloroplast genetic engineering, and related applications (Nyamgerel et al. 2023; Li et al. 2025; Jiang et al. 2025; Baasanmunkh, Tsegmed, et al. 2026; Karimov et al. 2026). To date, chloroplast genomes of 27 *Oxytropis* species have been sequenced and published worldwide (Su et al. 2019; Liu et al. 2021; Bei et al. 2022; Tavares et al. 2022; Hu et al. 2024; Li et al. 2025; Zhang et al. 2025). Li et al. (2025) provided a comparative plastome analysis and phylogenetic relationships based on the most widely available *Oxytropis* species.

In Mongolia, 100 *Oxytropis* species, including four endemic species, have been reported, belonging to five subgenera and 20 sections based on traditional classifications (Ulziikhutag 2003; Malyshev 2008; Baasanmunkh et al. 2021; Baasanmunkh et al. 2022). Recently, three new species, *O. oyunmaae* Munkht. & Baasanm. (section *Verticillares*; Baasanmunkh, Munkhtulga, Oyuntsetseg, and Choi 2025), *O. jamsranii* Munkht. & Baasanm. (section *Xerobia*; Baasanmunkh, Munkhtulga, Oyuntsetseg, Jeong, and Choi 2025), and *O. dariimaae* Munkht. & Baasanm. (section *Verticillares*; Munkhtulga et al. 2026) have been described from Mongolia based on morphological evidence. Additionally, *O. sobolevskajae* Pjak, first described from the Republic of Tuva, Russia, was recently discovered in the western part of Mongolia (Baasanmunkh, Munkhtulga, Oyuntsetseg, Jeong, and Choi 2025). During an intensive botanical exploration in Mongolia, an unidentified species of *Oxytropis* which was morphologically similar to the species of the section *Mesogaea*.

Section *Mesogaea* is comprised of 35 species based on morphological data, and represents a morphologically cohesive but phylogenetically complex lineage within the subgenus *Phacoxytropis* Bunge, distributed primarily in the Irano‐Turanian and Central Asian mountains (Vasilchenko et al. 1948; Grubov 2004; Malyshev 2008; Archambault and Strömvik 2012; Turdiev et al. 2025). Of these 35 species, only 8 species are characterized by having pale yellow to yellow flowers. A high concentration of species occur in the mountain systems of the Mongolian Altay, Tien Shan, Nanshan, and Himalayas. In addition, section *Mesogaea* includes species with wide distribution ranges, such as Eurasian ( *O. lapponica* ) (Wahlenb.) J.Gay, *O. pilosa* (L.) DC., North Asian ( *O. glabra* ), and even Asian–North American ( *O. deflexa* ) species. The North Tibetan–western Chinese group (*O. gueldenstaedtioides* Ulbr., *O. kansuensis* Bunge, *O. melanocalyx* Bunge, *O. ochrocephala* Bunge, *O. shennongjiaensis* D.G.Zhang, J.T.Chen, T.Deng & H.Sun) also represents a biogeographically significant component of the section (Grubov 2004; Ulziikhutag 2003). Species in this section are characterized by their dense blackish or mixed indumentum, free stipules, and distinctly pendulous legumes. These morphological characters are traditionally used to distinguish the section *Mesogaea* from related sections, such as section *Janthina* Bunge and section *Phacoxytropis* (Polozhii 1994; Malyshev 2008; Zhu et al. 2010). Despite this apparent morphological coherence, recent molecular studies using ITS, and plastid regions have demonstrated that *Mesogaea* is not monophyletic, with its constituent species scattered across multiple clades within the broader *Phacoxytropis* subgenus (Kholina et al. 2021; Lei et al. 2025). Several new species have been described within this section based on morphological and molecular analyses, including *O. iridum* Dickoré & Kriechb. (Dickoré and Kriechbaum 2006), *O. shennongjiaensis* D.G.Zhang, J.T.Chen, T.Deng & H.Sun (Chen et al. 2020), and *O. mandokhailii* N.Khan, A.Sultan & M.Rashid (Khan et al. 2023).

We describe a new species, *O. ikhbogdicus* Munkht., Baasanm. & H.J.Choi, based on both morphological and molecular evidence.

## 2 Materials and Methods

### 2.1 Field Expeditions

**FIGURE 1:**
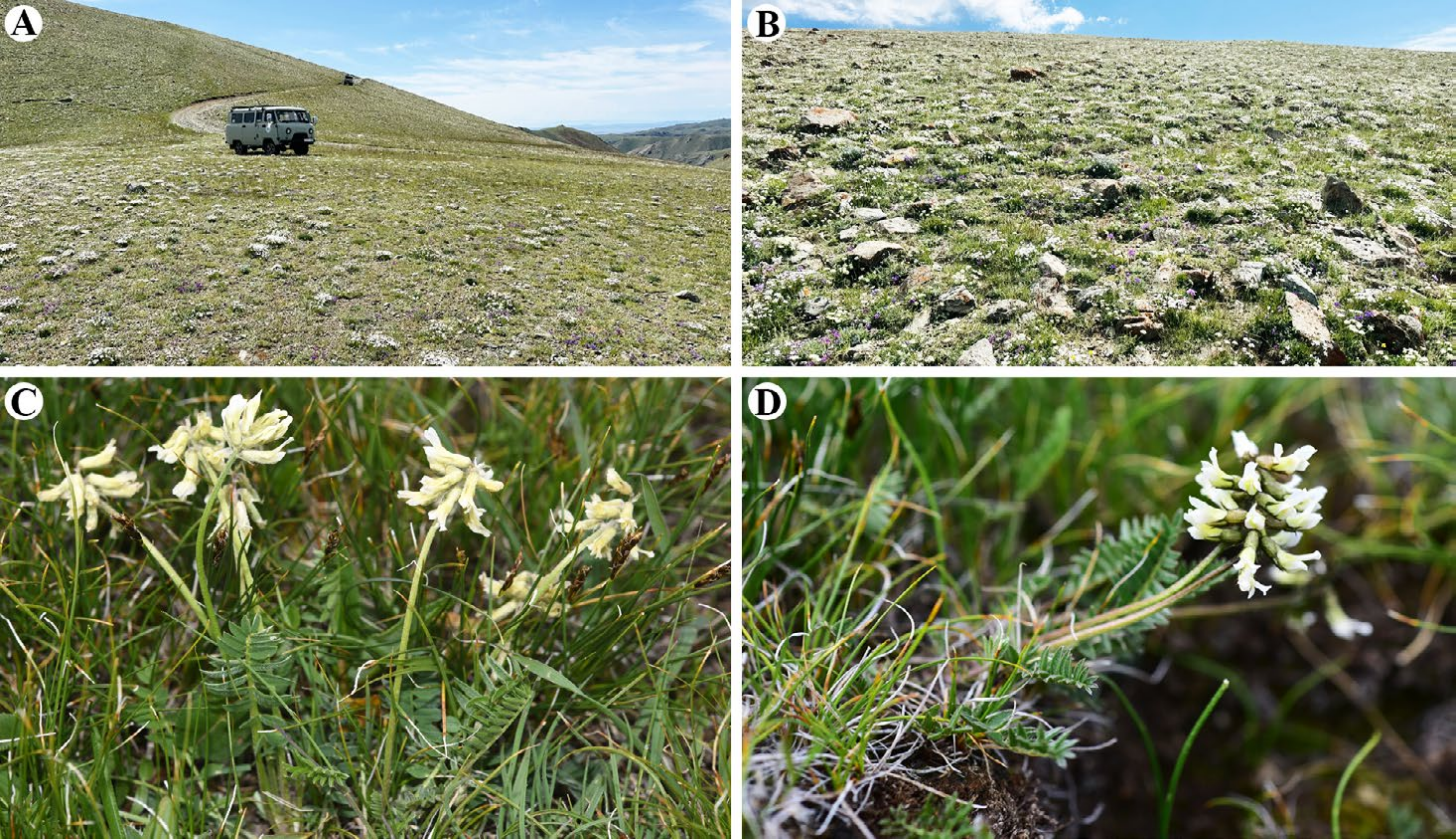
(A and B) General vegetation habitat of *Oxytropis ikhbogdicus* in the Ikh Bogd Mountain, Bayankhongor province. (C and D) Growth habits of *Oxytropis ikhbogdicus* (Photographs: S.Baasanmunkh and D.Munkhtulga).

Extensive field expeditions to collect *Oxytropis* samples, including herbarium specimens, fresh leaves, and detailed wild photographs of various vegetation types across Mongolia have been conducted by the authors since 2017. Over 250 herbarium specimens of *Oxytropis* were collected and deposited in the Herbarium of the National University of Mongolia (UBU). Field expeditions in the Ikh Bogd Mountains, Bayankhongor Province, took place during the period 02–14 July 2025. This mountain lies in the southern part of the country with a maximum elevation of 3600 m (Figure 1A). In addition, herbarium specimens from ALTB, LE, MW, NS, GFW, UBA, and UBU were examined (Thiers 2026) and FloraGREIF (https://floragreif.uni‐greifswald.de). Furthermore, photographic observation data of selected *Oxytropis* species from the “Flora of Mongolia” project were cross‐checked (https://www.inaturalist.org/projects/flora‐of‐mongolia; Baasanmunkh, Oyuntsetseg, et al. 2026) on the iNaturalist platform. A distribution map of the four species was produced using ArcGIS (Esri 2012).

### 2.2 Phylogenetic Studies

#### 2.2.1 Plant Material, DNA Extraction, and Sequencing

Fresh leaf materials of *O. ikhbogdicus* was collected and dried in silica gel from the type location on Ikh Bogd Mountain, Bayankhongor Province, Mongolia. Two voucher specimens were deposited in UBU with the following sheet barcodes: UBU0039445 and UBU0039446. Total genomic DNA was extracted from silica gel‐dried leaf material using the cetyltrimethylammonium bromide (CTAB) method (Doyle and Doyle 1987). DNA was purified with chloroform:isoamyl alcohol, precipitated with isopropanol, washed with ethanol, and resuspended in TE buffer. DNA quality and concentration were checked using a BioDrop spectrophotometer (Biochrom Ltd.). The library was constructed from genomic DNA using the TruSeq DNA Nano Kit and sequenced on the NextSeq 500 platform (Illumina, San Diego, CA, USA) according to the manufacturer's instructions. In addition, the nuclear ITS region (White et al. 1990) was amplified from genomic DNA by PCR. PCR amplification was performed using DNA template at a concentration of 100 ng/μL, following the protocol described previously (Munkhtulga et al. 2025). PCR products were sequenced in both directions by Macrogen (Seoul, Korea). DNA sequences were visually checked and manually trimmed in Geneious Prime 2024.0.7 (www.geneious.com).

#### 2.2.2 Genome Assembly, and Annotation

Trimmomatic software v.0.36 (Bolger et al. 2014) was employed to exclude adapter sequences and low‐quality reads to minimize bias. The overall quality of the sequencing data and quality score distribution across each cycle were assessed using FastQC v.0.11.5 (Antil et al. 2023). The complete chloroplast genome was *de novo* assembled from the filtered reads using NOVOplasty v.4.1.0 (Dierckxsens et al. 2017) with multiple k‐mer sizes to optimize assembly performance. Assembly and orientation were verified using the basic local alignment search tool (BLAST) program provided by the NCBI for Biotechnology Information (https://www.ncbi.nlm.nih.gov/). The annotation results were validated using the online software GeSeq Annotation of Organellar Genomes (Tillich et al. 2017). A circular map was created using OrganellarGenomeDRAW (OGDRAW; Greiner et al. 2019).

#### 2.2.3 Phylogenetic Analyses

To determine the phylogenetic position of the new species, we compared all available cp genomes and nuclear ITS sequences of the species within the section *Mesogaea* based on their morphological similarities. Additionally, we included several species from different sections and subgenera of *Oxytropis* and three species of *Astragalus* L. were obtained from the NCBI database (Table S1). The entire cp genome was aligned using MAFFT v.7.38871 (Katoh et al. 2002), and ITS sequences were aligned using MUSCLE v.5 (Edgar 2022). Phylogenetic trees were constructed using Bayesian Inference (BI) approaches. The BI analysis was conducted using MrBayes v.3.2.6 (Ronquist et al. 2012). Two separate runs were performed, each of which consisted of four chains running simultaneously. The analysis was performed for 5,000,000 generations using the Markov chain Monte Carlo (MCMC) algorithm. Tree samples were collected at intervals of 5000 generations, with the initial 25% of samples being excluded as burn‐in. The reconstructed trees were generated using FigTree v.1.4.4 (Rambaut 2012).

## 3 Results and Discussion

### 3.1 Assembly and Characteristics of the Chloroplast Genome

**FIGURE 2:**
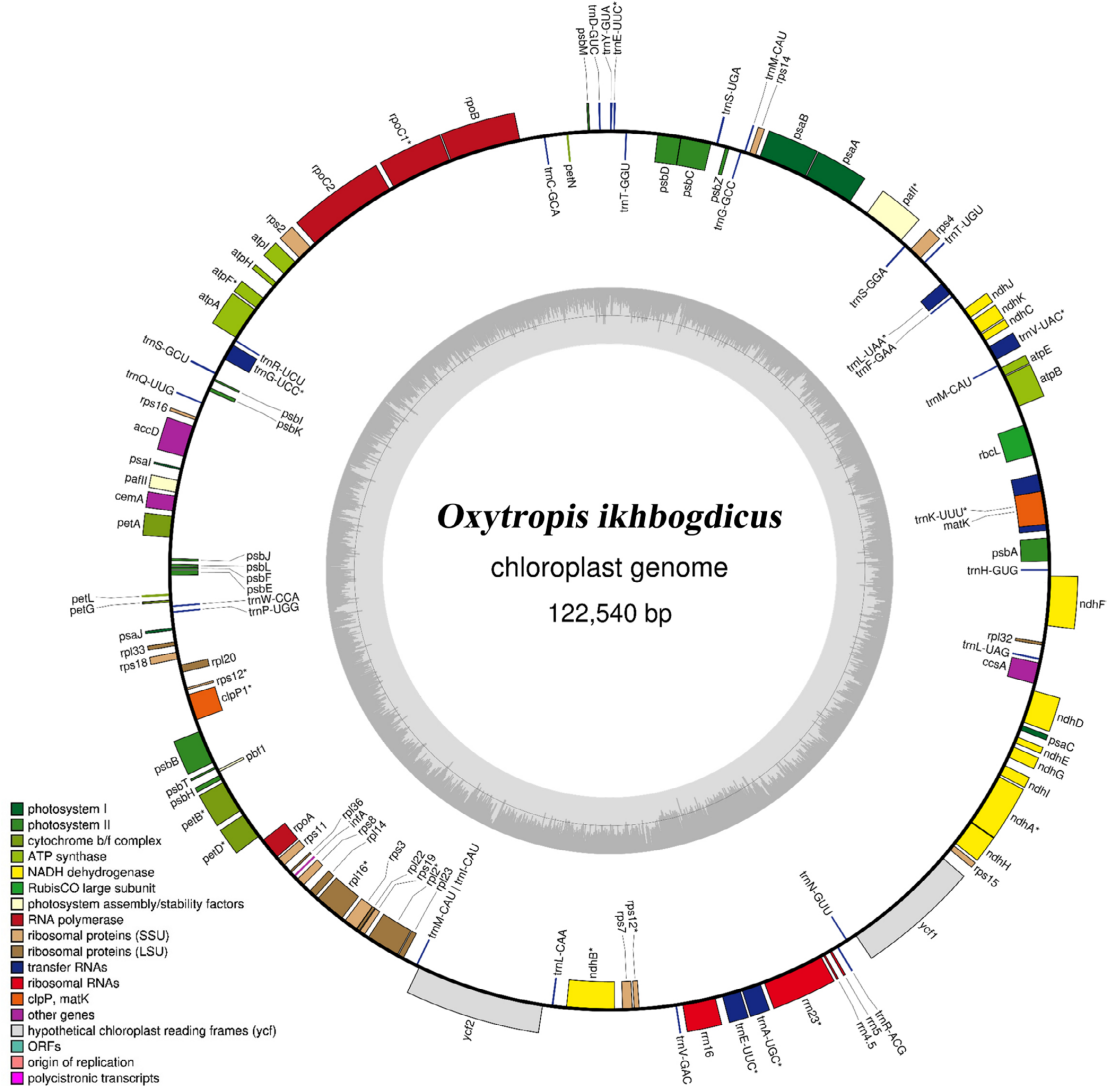
Circular gene map of the complete chloroplast genome of *Oxytropis ikhbogdicus*. Genes drawn inside the circle are transcribed clockwise, and those outside, counterclockwise. The function of genes is color‐coded. The darker gray in the inner circle represents GC content and light gray represents AT content.

The complete cp genome of *O. ikhbogdicus* was sequenced and the annotated genome was uploaded to GenBank with accession number PX652196. A total of 9.4 Gb of data comprising 62,284,714 paired‐end reads (150 bp) were produced. In total, 5,962,751 (9.57%) paired reads were mapped for assembly after cutting and normalization. A single contig with full coverage and an average mapping depth of 5980.05 was generated by *de novo* assembly. The cp genome of *O. ikhbogdicus* was a double‐stranded circular DNA of 122,540 bp in length. It contained a long single‐copy (LSC) region of 80,425 bp, a short single‐copy (SSC) region of 19,020 bp, and only one inverted repeat (IRs) of 23,155 bp (Figure 2). While the GC content in the LSC, SSC, and IR regions was 33.4%, 30.0%, and 40.0%, respectively, the total genomic GC content was 34.2%. This triad structure was preserved in the Papillonoideae owing to the loss of one IR and was referred to as the IR‐lacking clade (IRLC) (Wojciechowski et al. 2004; Jansen et al. 2008). The cp genome contained 110 genes, including 76 protein‐coding genes, four rRNA genes, and 30 tRNA genes (Figure 2). The newly sequenced species shared the same gene content as *Oxytropis* species, such as the loss of the whole genes of *inf*A, *rpl*22, and *rps*16, and one intron of *atp*F, *clp*P1, and *rps*12 (Bei et al. 2022; Su et al. 2019; Tavares et al. 2022; Lei et al. 2025; Li et al. 2025). Among the protein‐coding genes, nine contained introns, eight of which (*clp*P1, *ndh*A, *ndh*B, *pet*B, *pet*D, *rpl*16, *rpl*2, and *rpo*C1) contained two exons and one intron each, whereas the *ycf*3 had three exons and two introns. The *rps*12 gene was a trans‐spliced gene with the 5′‐end located in the LSC and the 3′‐end located in an IR region. In addition, six tRNAs each contained two exons and one intron. The longest and shortest introns were present in tRNAs, namely *trn*K‐UUU with 2489 bp and *trn*L‐UAA with 532 bp. The genes and their lengths in the cp genome of *O. ikhbogdicus* were similar to those of other species of *Oxytropis* (Lei et al. 2025; Li et al. 2025).

### 3.2 Phylogenetic Analysis

Two datasets (cp genome and ITS) were compiled to verify the phylogenetic position of *_O. ikhbogdicus_*. The cp genome dataset included 37 aligned genomes with a total length of 148,155 bp, of which 53,632 bp (36.2%) were variable, and 11,112 bp (7.5%) were parsimony‐informative. The ITS dataset included 63 aligned sequences with a length of 595 bp, of which 124 characters (20.8%) were variable and 10 characters (1.6%) were parsimony‐informative. Both datasets showed that the outgroup species were robustly separated from the *Oxytropis* species, supporting previous studies that demonstrated the monophyly of the *Oxytropis* genus (Shavvon et al. 2017; Tekpinar et al. 2016a, 2016b; Li et al. 2025).

**FIGURE 3:**
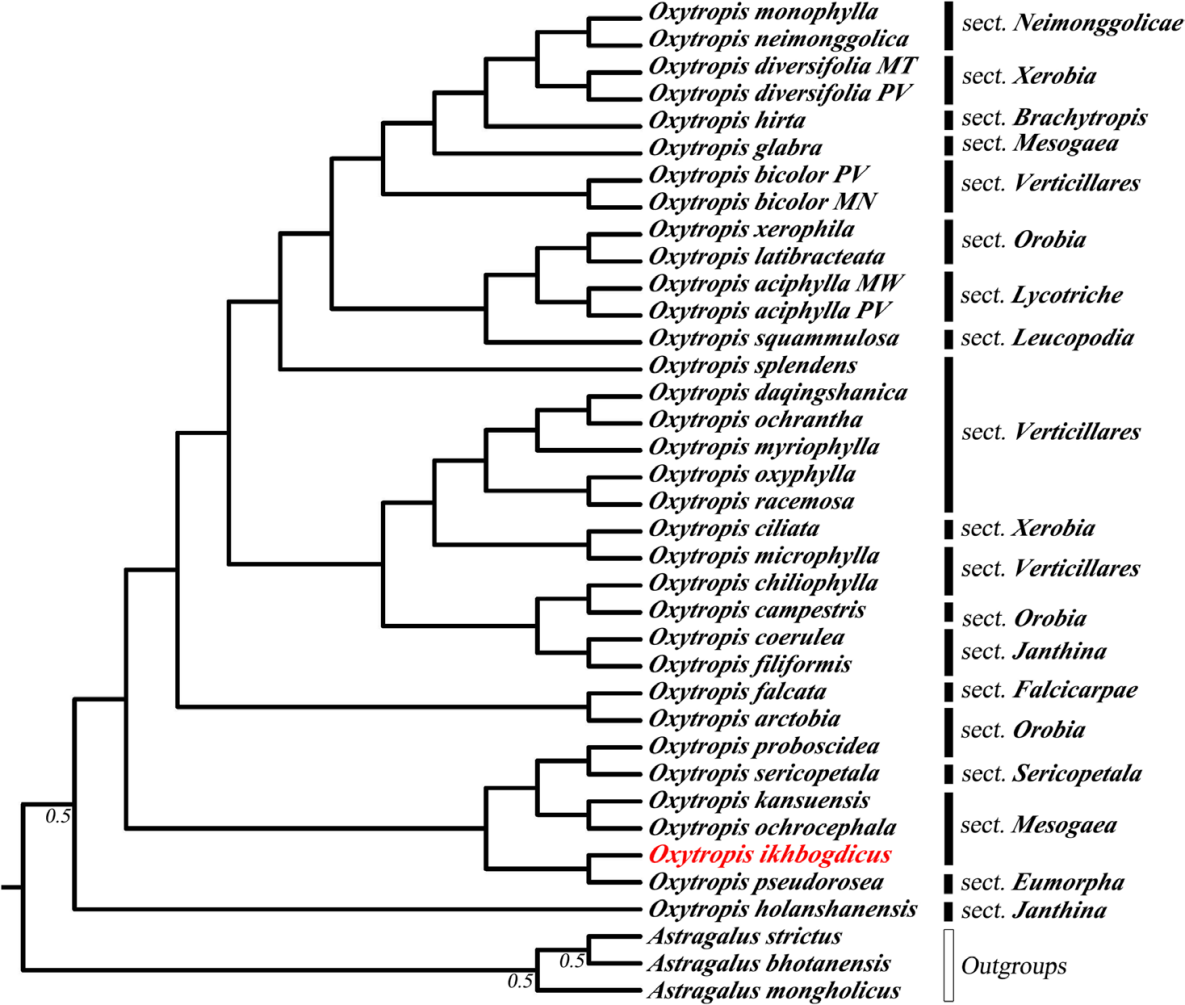
The phylogenetic tree of the *Oxytropis* species was constructed by Bayesian inference based on entire chloroplast genome sequences. Posterior probability (PP) values are shown at the nodes; values of 1.0 are not displayed on the phylogenetic tree. New species (*Oxytropis ikhbogdicus*) marked in the red color.

**FIGURE A1:**
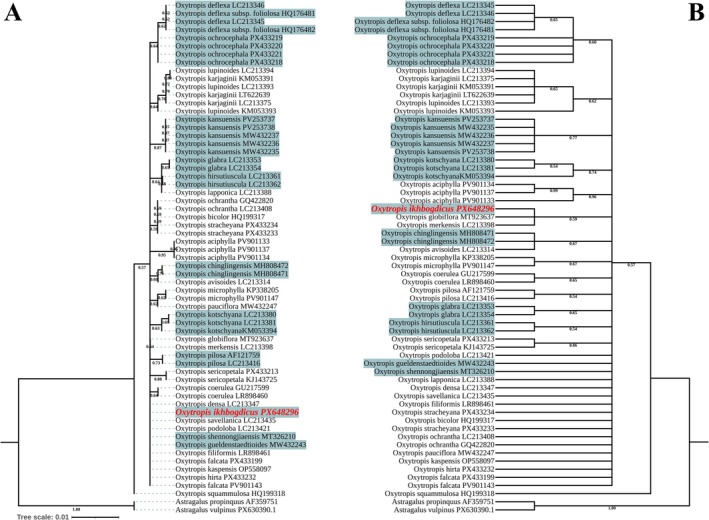
Phylogenetic tree of *Oxytropis* species based on ITS sequences. (A) Neighbor‐Joining (NJ) tree. (B) Maximum Parsimony (MP) tree. Bootstrap support values (> 50%) are indicated at the branch nodes. The newly described species is highlighted in bold red, while species belonging to section *Mesogaea* are marked with a green background.

Incongruence was observed between the cp genome (Figure 3) and ITS phylogenies (Figure A1) across the majority of branches. It may reflect differences in evolutionary history, such as incomplete lineage sorting or potential hybridization events (Liu et al. 2025), which have been widely reported in Fabaceae (Xu et al. 2012; Zhou et al. 2023; Fang 2024; Zhang et al. 2026). As one of the most complex and polymorphic genera in Fabaceae, *Oxytropis* has been the focus of numerous studies to clarify the phylogenetic relationships among its species. Jorgensen et al. (2003) reported that sequence divergence was very low and ranged from 0% to 1.48% among the *Oxytropis* taxa. Similarly, Archambault and Strömvik (2012) found that ITS sequences among 81 *Oxytropis* species are highly similar, with interspecific divergence almost five times lower than in *Astragalus*. Consistent with these findings, our ITS dataset showed less than 2% interspecific variation between *Oxytropis* species (Table S2). The low interspecific divergence observed in ITS sequences suggests that this marker has limited discriminatory power for resolving species boundaries within *Oxytropis* (Figure A1).

**FIGURE A2:**
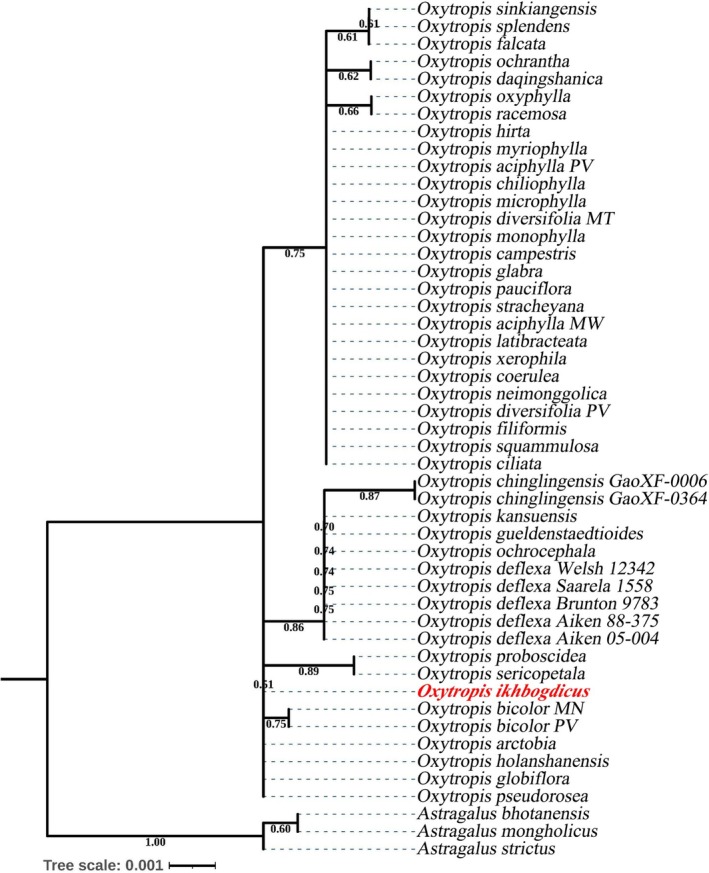
Phylogenetic tree of *Oxytropis* species based on combined *mat*K and *rbc*L sequence data inferred using the Neighbor‐Joining (NJ) method. Bootstrap support values (> 50%) are indicated at the nodes. The newly described species is highlighted in bold red.

Previous phylogenetic studies of *Oxytropis* have widely employed chloroplast markers such as *trn*H–*psb*A, *trn*L intron, *trn*L–F, *and trn*V intron, *trn*S–*trn*G, and *pet*G–*trn*P (Kholina et al. 2021; Tekpinar et al. 2016a; Tekpinar et al. 2016b). The variability of these chloroplast markers have provided useful resolution for phylogenetic relationships among certain species and sections within *Oxytropis*. However, the relatively limited variation of these individual markers often restricts their ability to fully resolve deeper and more complex phylogenetic relationships within the genus. To further assess marker performance in our dataset, we compared pairwise sequence identity between the most variable conventional chloroplast barcode dataset (*mat*K + *rbc*L) and cp genome sequences across all available taxa within the section *Mesogaea*. Phylogenetic analysis based on the *mat*K + *rbc*L dataset showed limited resolution among closely related *Oxytropis* species, with several taxa forming weakly supported or unresolved relationships (Figure A2). In contrast, the cp genome dataset exhibited substantially greater interspecific sequence divergence than the barcode dataset (Tables S3 and S4), indicating a higher discriminatory power for resolving species‐level relationships within the genus *Oxytropis*. Our findings are consistent with recent comparative phylogenetic analyses, which demonstrated that complete plastid genome sequences provide superior phylogenetic resolution compared with conventional molecular markers (Li et al. 2025).

Although *O. ikhbogdicus* is morphologically similar to *O. kansuensis* and *O. ochrocephala* of the section *Mesogaea*, phylogenetic analysis based on complete chloroplast genome sequences recovered it as sister to *O. pseudorosea* of the section *Eumorpha* (Figure 3). This difference between traditional sectional classification and molecular phylogeny suggests that morphological characters currently used for sectional delimitation in *Oxytropis* may reflect convergent evolution or taxonomically unstable characters.

## 4 Taxonomic Treatment

**FIGURE 4:**
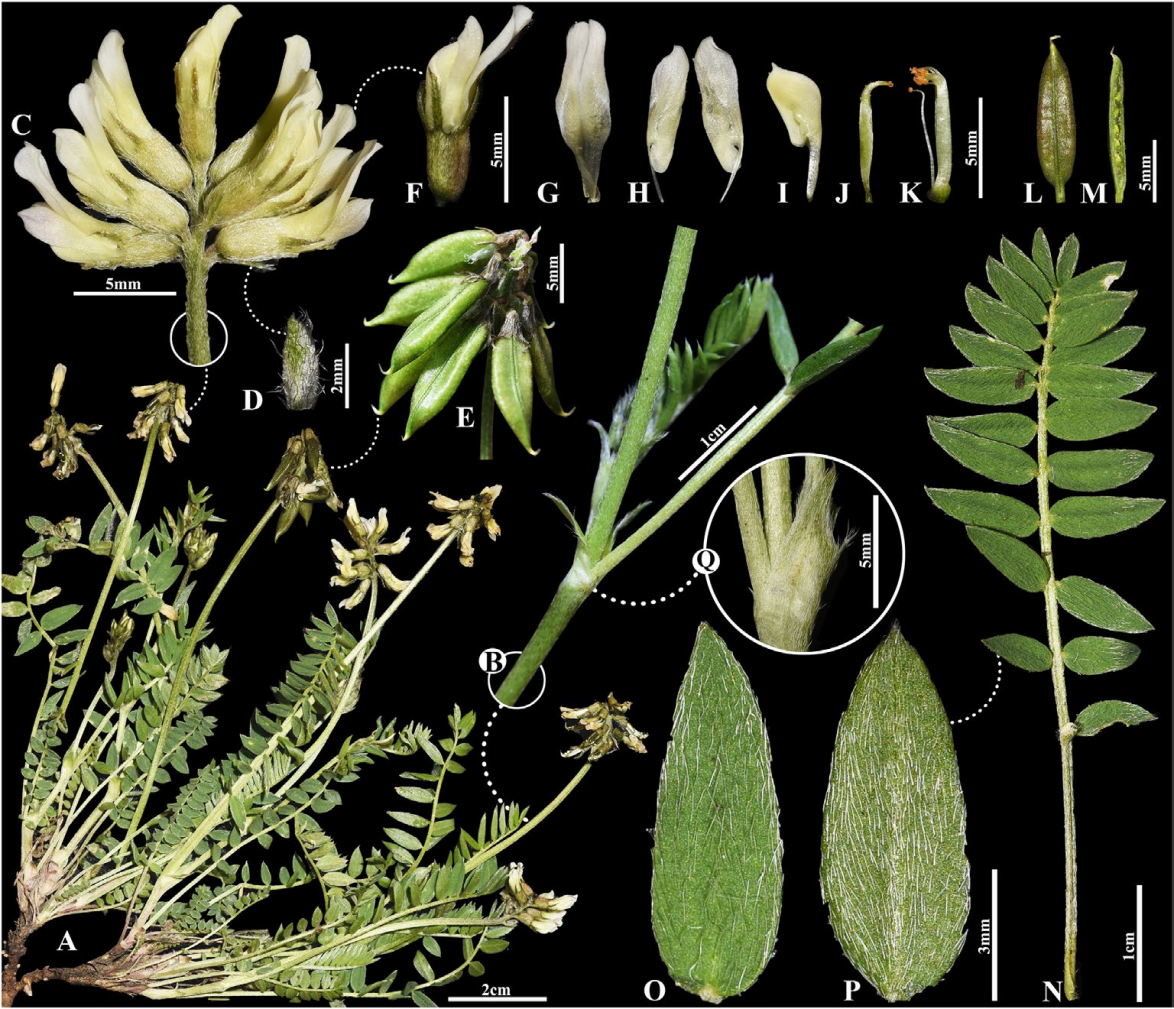
*Oxytropis ikhbogdicus* Munkht., Baasanm. & H.J.Choi in Mongolia. (A) General habit, (B) Stem, (C) Raceme, (D) Bract, (E) Pods, (F) Flower, (G) Standard, (H) Wings, (I) Keel, (J) Pistil, (K) Stamens, (L) Pod, (M) Pod valve, (N) Leave, (O) Leaflet, adaxial view, (P) Leaflet, abaxial view, (Q) Stipule (Photographs: D.Munkhtulga).

**FIGURE 5:**
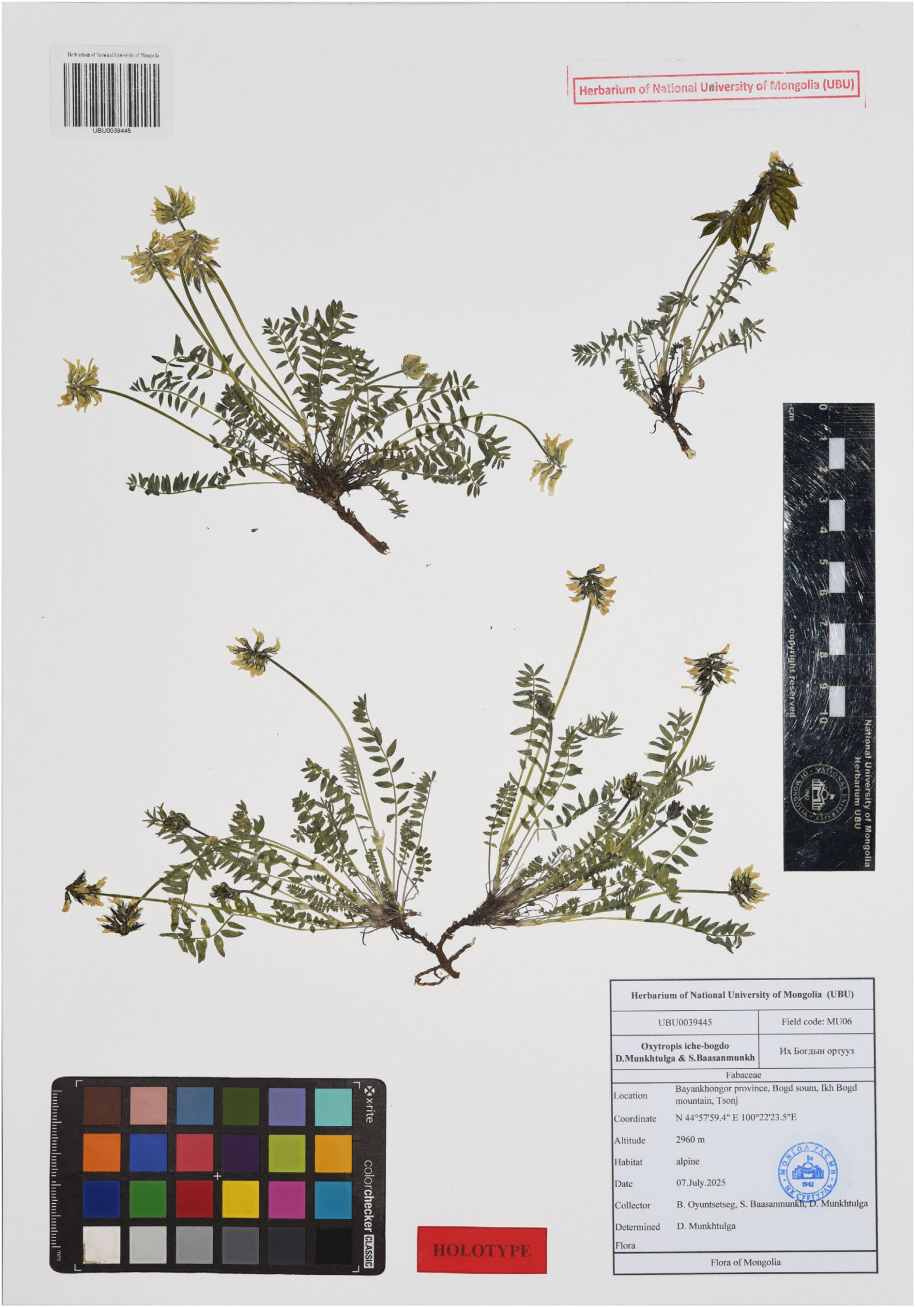
Photograph of the holotype of *Oxytropis ikhbogdicus* Munkht., Baasanm. & H.J.Choi.

***Oxytropis ikhbogdicus*** Munkht., Baasanm. & H.J.Choi, sp. nov. (Figures 4 and 5).

**Type**

MONGOLIA. Bayankhongor Province, Bogd soum, Ikh Bogd mountain, 44.966512 N, 100.37319 E, 2960 m, 07 July 2025, B. Oyuntsetseg., S. Baasanmunkh., D. Munkhtulga., MU06 (holotype: UBU‐0039445; isotype: UBU‐0039446).

**Diagnosis**

**FIGURE 6:**
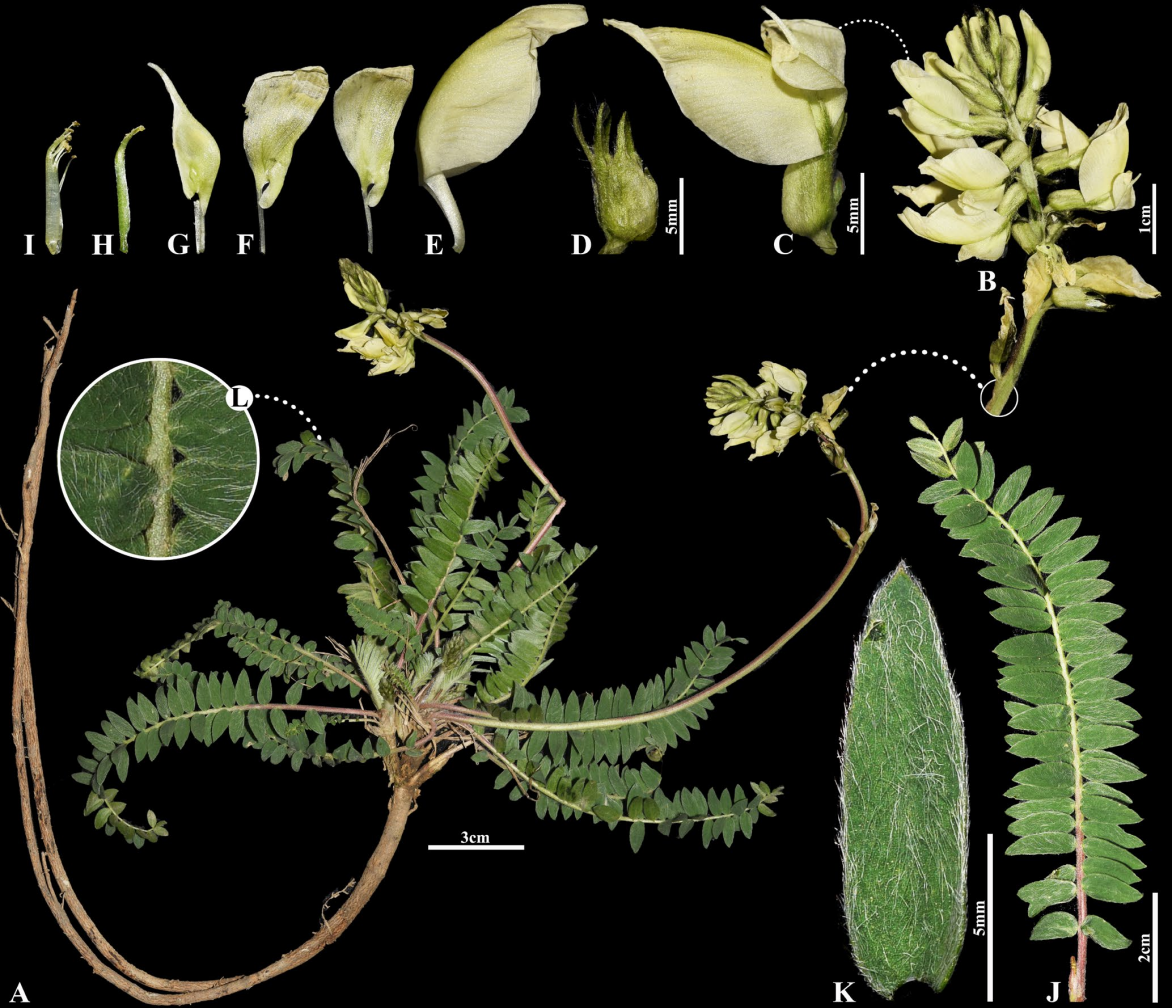
*Oxytropis ochroleuca* in Kazakhstan. (A) General habit, (B) Raceme, (C) Flower, (D) Calyx, (E) Standard, (F) Wings, (G) Keel, (H) Pistil, (I) Stamens, (J) Leave, (K) Leaflet, (L) Leaflets pubescent (Photographs: D.Munkhtulga).

*Oxytropis ikhbogdicus* (Figure 4) is morphologically most similar to *O. kansuensis*, *O. ochroleuca* Bunge (Figure 6) and *O. ochrocephala* , within section *Mesogaea* but differs by its stems with 1–2 conspicuous internodes (vs. with 2–5 conspicuous internodes in *O. kansuensis*, *O. ochroleuca*, and *O. ochrocephala* ); calyx campanulate, 6–7 mm long (vs. campanulate, 6.5–11.5 mm long in *O. kansuensis*; broadly cylindric, 7–8 mm long in *O. ochroleuca*; cylindric, 6–8 mm long in *O. ochrocephala* ); and standard 8.5–9.0 mm long, lamina rhombic‐oblanceolate, apex rounded to emarginate (vs. 10–17 mm long, lamina ovate, apex emarginate in *O. kansuensis*; 12–16 mm long, lamina orbicular‐ovate, apex 2‐lobed in *O. ochroleuca*; 10–17 mm long, lamina broadly obovate, apex emarginate in *O. ochrocephala* ) (Table 1).

**TABLE 1:** Morphological comparisons of four *Oxytropis* species in section *Mesogaea*.

Characters	*O. ikhbogdicus*	*O. ochroleuca*	*O. ochrocephala*	*O. kansuensis*
Plant height	10–15 cm	5–30 cm	10–56 cm	12–40(−60) cm
Branches	Stems with 1 or 2 conspicuous internodes.	Stems with 2–4 conspicuous internodes.	Stems with 2–5 conspicuous internodes.	Stems with (3 or) 4 or 5 conspicuous internodes
Leaves	6–8 cm long, leaflets 12–15 pairs; petiole and rachis with appressed‐pilose	5–15(−19) cm long, leaflets 10–17 pairs; petiole and rachis with spreading white pilose	3–19 cm long, leaflets 6–13(−20) pairs; petiole and rachis with long yellow pilose and pale brown glands	(2.5–)4–14(−20) cm long, leaflets 8–15(−18) pairs; petiole and rachis with glabrescent to sparsely spreading white villous
Racemes	8–12‐flowered; peduncles 8–10 cm long	6–14‐flowered; peduncle 5–12 cm long	8–14‐flowered or more; peduncle (3.5–)10–25 cm long	3–15‐flowered; peduncle 7–21(−30) cm long
Calyx	Campanulate, 6–7 mm	Broadly cylindric, 7–8 mm	Cylindric, 5.8–7.2(−8.5) mm	Campanulate, 6.5–11.5 mm
Flowers	Yellow to white; standard 9 × 3.0–3.5 mm, lamina rhombic‐oblanceolate, apex rounded to emarginate	Pale yellow; standard 12–16 × 4–5 mm, lamina orbicular‐ovate, apex 2‐lobed	Yellow; standard 10–17 × 8–11 mm, lamina broadly obovate, apex emarginate	Yellow or pale yellow; standard 10–17 × 6–8 mm long, lamina ovate, apex emarginate
Pod	Oblong‐ellipsoid, 13–16 mm long, membranous	Cylindric‐ovoid, (10–)15–25 mm long, membranous	Oblong, 12–15 mm long, leathery	Oblong‐ovoid, 8–12 mm long, papery
Distribution	Mongolia	China (Xinjiang), Kazakhstan, Kyrgyzstan	China (North‐Central, China South‐Central, Qinghai, Tibet)	China (Gansu, Qinghai, Sichuan, Xizang)

**Description**

*Plants* perennial, herbs, 10–15 cm tall, caulescent, arising from a multi‐headed caudex. Stems erect or sprawling, with 1–2 apparent internodes, with appressed pilose. *Stipules* lanceolate or ovate‐lanceolate, herbaceous, with densely pilose, connate as a sheath, apex acute. *Leaves* imparipinnate 6–8 cm long; petiole 2–3 cm long; petiole and rachis with appressed pilose. *Leaflets* 12–15 pairs, blades oblong‐lanceolate to broadly elliptic 6–9 × (2.5–)3.0–3.5 mm, sub‐obtuse to acute at the apex, both surfaces appressed pilose. *Racemes* at first dense, becoming lax, 8–12‐flowered; peduncles 8–10 cm long, erect, as long as or longer than leaves, strigose. *Bracts* lanceolate, 2.5–3.0 × 1.0–1.5 mm, membranous, with sparse, white and black trichomes intermixed. *Calyx* campanulate, 6–7 × 2.5–3.0 mm, densely covered with appressed white sericeous, sometimes mixed with appressed black short trichomes; teeth subulate, ca. 2.5 mm long. *Corolla* glabrous, cream to pale yellow; standard ca. 9 × 3.0–3.5 mm, lamina rhombic‐oblanceolate, apex rounded to emarginate, basally tapering into claw; wings 7.0–7.5 × ca. 2.5 mm, lamina oblong, apex rounded, claw ca. 2.5 mm long; keel ca. 6.5 × ca. 2.5 mm, claw ca. 2.5 mm long, beak ca. 0.2 mm long. Stamens diadelphous, with nine filaments connate, one filament free. Ovary linear, ca. 5 mm long, with densely appressed‐pilose, subsessile; style glabrous, shorter than ovary, curved at apex. *Pods* stipitate (stipe ca. 1.5 mm long), oblong‐ellipsoid, 13–16 × 3.5–4.0 mm, pendulous, membranous, slightly inflated and flattened, with sparsely appressed short black trichomes, apex with a beak.

**Phenology**

Flowering in June and July. Fruiting from late July and early August.

**Etymology**

The specific epithet *ikhbogdicus* refers to the habitat of this species in the Ikh Bogd Mountain of Mongolia.

**Distribution and Habitat**

**FIGURE 7:**
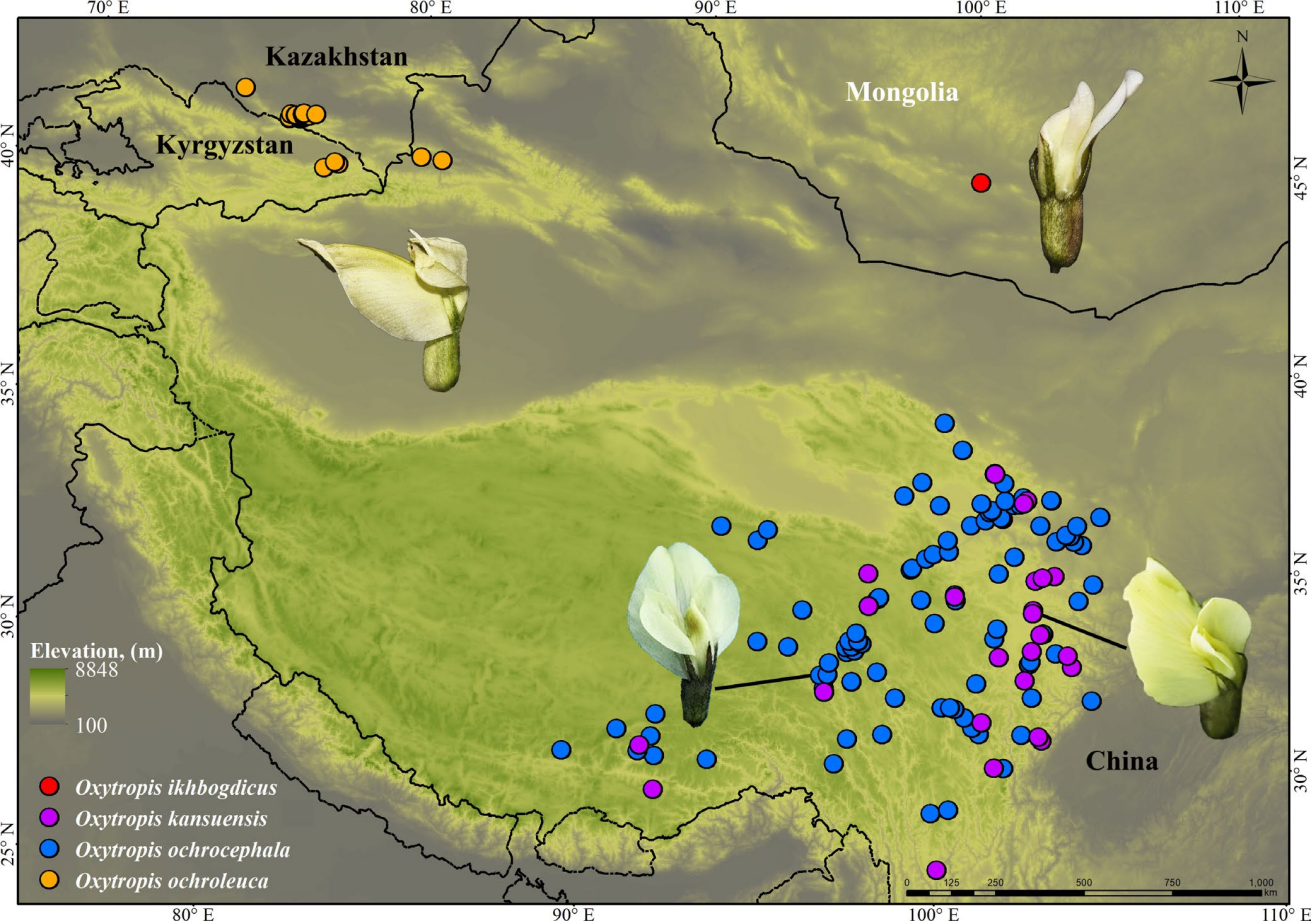
Distribution map of four *Oxytropis* species in Asia. *Oxytropis ikhbogdicus* (red circle), *O. ochrocephala* (blue circle), *O. ochroleuca* (orange circle), *O. kansuensis* (purple circle).

*Oxytropis ikhbogdicus* is currently known only from the Ikh Bogd Mountain in Bayankhongor Province, Mongolia. Given the substantial distance from that of neighboring countries, we consider this species to be endemic to Mongolia (Figure 7). It grows on stony slopes, damp meadow plots, *Carex*‐*Kobresia* vegetation, and riverbanks in the alpine belt, at 2800–3500 m a.s.l. (Figure 1).

**Preliminary Conservation Status**

During our expeditions, we found only two isolated populations in the Ikh Bogd Mountain areas. Therefore, we propose that it be listed under the Data Deficient (DD) category following the guidelines of IUCN Standards and Petitions Committee (2024).

## 5 Conclusion

Globally, the taxonomic identification of species within the genus *Oxytropis* is challenging, given the extensive species diversity and complex morphological characters. In this study, we describe a new species of *Oxytropis* from the Ikh Bogd Mountain in Mongolia based on its morphological and molecular evidence. We found that the chloroplast genome dataset exhibited substantially greater interspecific sequence divergence than the barcode markers, indicating a higher discriminatory power for resolving species‐level relationships within the genus *Oxytropis*.

## Author Contributions

**Dariganga Munkhtulga:** conceptualization (equal), data curation (equal), formal analysis (lead), investigation (equal), methodology (equal), resources (equal), software (equal), writing – original draft (equal). **Shukherdorj Baasanmunkh:** conceptualization (equal), data curation (equal), investigation (equal), methodology (equal), resources (equal), supervision (equal), validation (equal), visualization (equal), writing – original draft (equal), writing – review and editing (equal). **Nudkhuu Nyamgerel:** formal analysis (lead), investigation (equal), methodology (equal), resources (equal), software (equal), writing – original draft (equal). **Batlai Oyuntsetseg:** data curation (equal), investigation (equal). **Zagarjav Tsegmed:** software (equal). **Dong Pil Jin:** investigation (equal), writing – review and editing (equal). **Serik Kubentayev:** investigation (equal), writing – review and editing (equal). **Gun‐Aajav Bayarmaa:** project administration (equal). **Hyeok Jae Choi:** conceptualization (equal), project administration (lead), supervision (equal), writing – review and editing (equal).

## Funding

This study was supported by Korea Basic Science Institute (2023R1A6C101B022), National University of Mongolia (P2024‐4834), Sejong National Arboretum (2024‐KS‐OB‐1‐1‐1‐16).

## Ethics Statement

The authors have nothing to report.

## Conflicts of Interest

The authors declare no conflicts of interest.

## Supporting information


**Table S1:** Voucher information and GenBank accession numbers for sequences used in this study.
**Table S2:** Identity (%) of the ITS sequence among the *Oxytropis* species.
**Table S3:** Identity (%) of the complete chloroplast genome sequence among the Oxytropis species.
**Table S4:** Identity (%) of the concatenated matK and RbcL sequence among the Oxytropis species.

## Data Availability

The DNA sequences generated in the present study have been deposited in the National Center for Biotechnology Information (NCBI) database. The accession numbers and the information on the voucher specimens are available in Table S1. The voucher specimens of the new species were housed in UBU.
